# Supplementary material for: On-chip generation and dynamic piezo-optomechanical rotation of single photons
Source: Nat Commun. 2022 Nov 16;13:6998. doi: 10.1038/s41467-022-34372-9 (PMC9668908; doi:10.1038/s41467-022-34372-9)
Supplement: Supplementary file 1 — Supplementary Information [file 41467_2022_34372_MOESM1_ESM.pdf]

Supplementary Information

On-chip generation and dynamic piezo-optomechanical rotation of single photons

Dominik D. Bühler, Matthias Weiß, Antonio Crespo-Poveda, Emeline D. S. Nysten, Jonathan J. Finley, Kai Müller, Paulo V. Santos, Mauricio M. De Lima Jr., Hubert J. Krenner

Table of contents

Supplementary Note 1 – Device component design
- **Supplementary Note 1.1 – Integrated quantum photonic circuit design**
- **Supplementary Note 1.2 – Interdigital transducers**
- **Supplementary Note 1.3 – Sample micrographs**

Supplementary Note 2 – Experimental setup

Supplementary Note 3 – Sample characterization
- **Supplementary Note 3.1 – Waveguide loss**
- **Supplementary Note 3.2 – SAW characterization**
- **Supplementary Note 3.3 – Passive MZI coupling**
- **Supplementary Note 3.4 – Simulation of the MZI switching dynamics**
- **Supplementary Note 3.5 – Acousto-optic spectral tuning of QDs**
- **Supplementary Note 3.6 – Temperature dependent effects**

Supplementary Note 4 – Second order autocorrelation function experiments
- **Supplementary Note 4.1 – Second order autocorrelation function of unmodulated QD**
- **Supplementary Note 4.2 – Intensity modulation of the two QDs selected for $g^{(2)}$ experiments**
- **Supplementary Note 4.3 – Fit functions**

Supplementary Note 5 – Wavelength selective routing: complementary measurements and analysis

Supplementary References

Supplementary Note 1 – Device component design

Supplementary Note 1.1 – Integrated quantum photonic circuit design

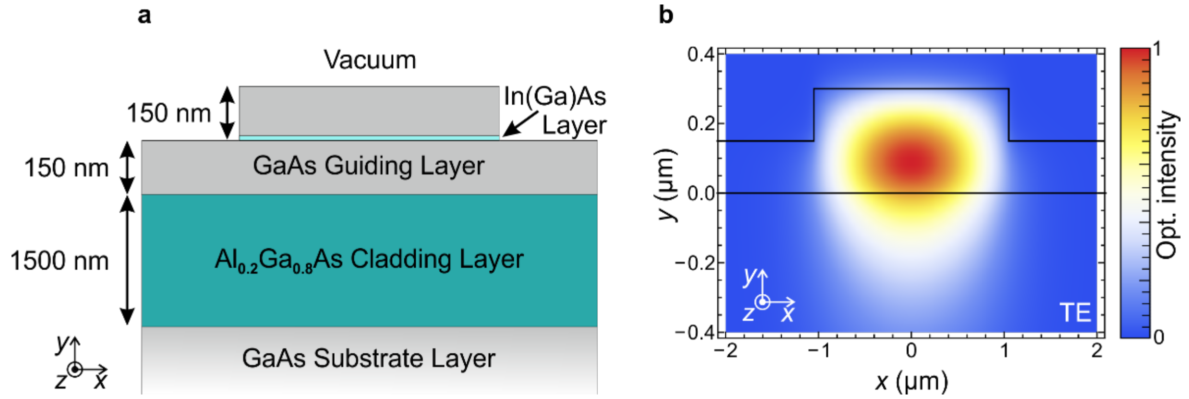

Supplementary Figure 1 – Ridge waveguide cross section - (**a**) Sketch of the waveguide cross section displaying the material layer sequence with thicknesses as indicated to the left. (**b**) Simulation of the fundamental TE mode confinement within the ridge waveguide structure.

The layer sequence of the sample is displayed in a schematic of the ridge waveguide cross-section in Supplementary Figure 1a. The layer sequence was as follows: first, a 1500 nm thick $Al_{0.2}Ga_{0.8}As$ cladding layer is grown on a (001)-GaAs substrate. The waveguiding layer consists of a 300 nm thick GaAs layer with a layer of (In,Ga)As QDs in its center. As shown Supplementary Figure 1a, the nominal etch depth to define the ridge waveguides was 150 nm. Supplementary Figure 1b shows the optical field distribution of the fundamental TE mode within the GaAs guiding layer (thick lines). The calculation assumes vacuum as the topmost layer and $Al_{0.2}Ga_{0.8}As$ as cladding. The waveguide itself is 2.1 µm wide. The effective refractive index n_{eff} can be calculated with the effective index method[1]. For this we used the refractive indices of the III-V compounds and their temperature dependencies reported in references[2–4].

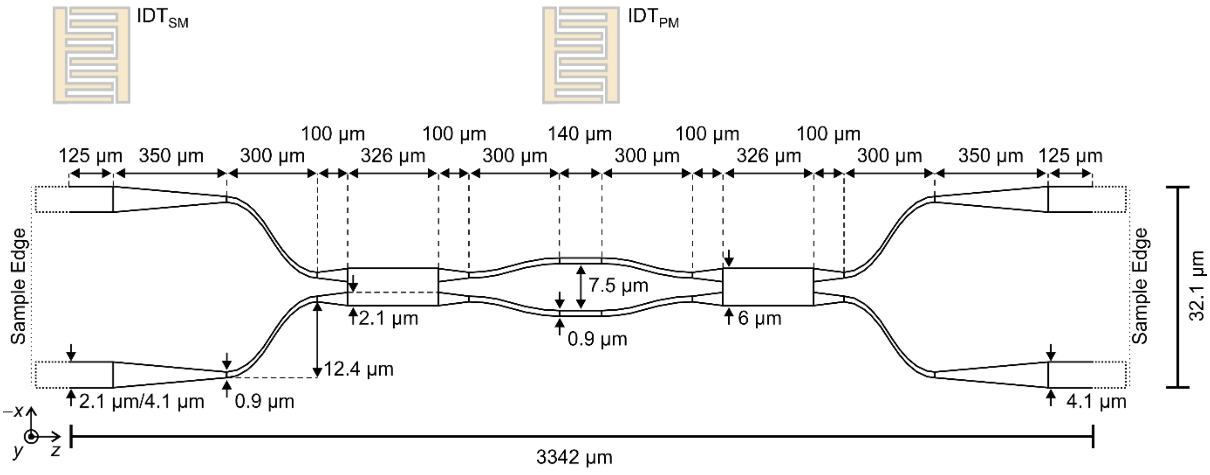

Supplementary Figure 2 – IQPC design –Full IQPC layout with all design parameters labeled.

Supplementary Figure 2 shows the full design of the IQPC with the dimensions labeled. In addition, the positions of the IDTs to excite SAWs are indicated (size of the IDTs not to scale). The design is 3342 µm long (*z*) and 32.1 µm (*x*) wide. The IQPC comprises output waveguides, tapers, S-bends, multimode interference (MMI) couplers, which form an integrated Mach-Zehnder interferometer (MZI). The circuit is designed for optimum SAW modulation[5]. Key design parameters were as follows: the MMI

lengths are chosen according to $L_{\mathrm{MMI}} = 3/2 L_\pi$, with L_π being the beat length[6], given by $L_\pi \approx \frac{4\, n_{\mathrm{eff}} W_{\mathrm{e}}}{3\lambda}$. Here, W_{e} is the effective waveguide width, taking into account the Goos-Hänchen shift[7]. Furthermore, it is important to note that the center of the MZI arms in the acousto-optic region are separated by $(2m+1)\Lambda_{\mathrm{SAW}}/2$ (m integer, Λ_{SAW}, SAW wavelength) along x-direction. For maximum modulation contrast[8] and the most compact design we set $m = 1$. In the MZI section, another important parameter to consider are the waveguide width, W compared to Λ_{SAW}. Here, the ratio is $\frac{W}{\Lambda_{\mathrm{SAW}}} = \frac{0.9\ \mu\mathrm{m}}{5.6\ \mu\mathrm{m}} \approx 0.16$, ensuring a well-defined modulation of the refractive index in each arm that can be safely assumed to be constant across the waveguide cross-sections.

Supplementary Note 1.2 – Interdigital transducer design

The IDTs used in the experiments presented had a Split-2 finger design to suppress internal reflections. The design wavelength was $\Lambda_{\mathrm{SAW}} = 5.6\ \mu\mathrm{m}$ (corresponding to a frequency of 520 MHz). The IDTs consist of 1400 fingers (aperture = 120 µm) distributed evenly over a total length of 1960 µm.

Supplementary Note 1.3 – Sample micrographs

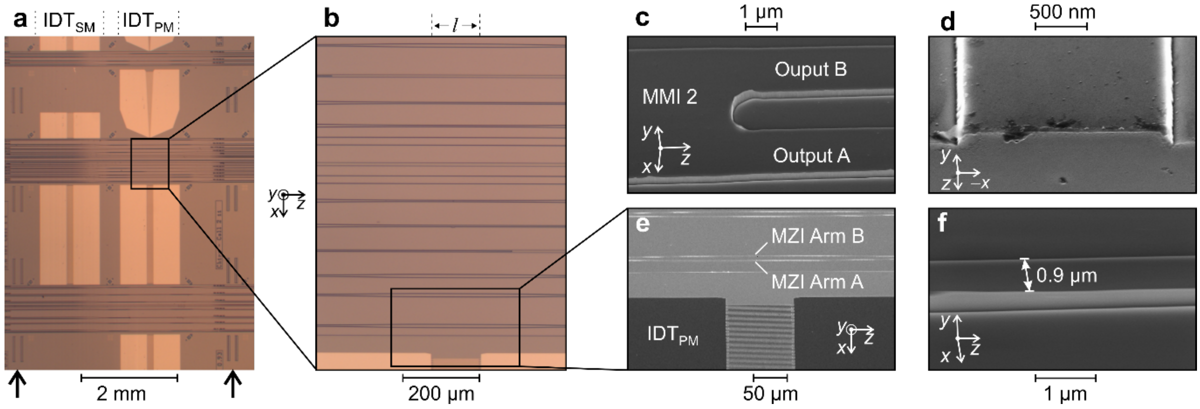

Supplementary Figure 3 – Sample micrographs – (**a**) and (**b**) are micrograph images showing part of the fully processed wafer before cleaving. SEM images of the outputs of the second MMI (**c**), the WG cross-section (**d**), $\mathrm{IDT_{PM}}$ (**e**) and a MZI arm WG (**f**) of a typical device.

Supplementary Figure 3 shows micrographs of the fabricated devices. Supplementary Figures 3a and 3b show two optical microscope images of one rectangular sample cell on the wafer before cleaving and mounting. The IDTs in the left-hand column of Supplementary Figure 3a can be used as the spectral modulators, $\mathrm{IDT_{SM}}$. The IDTs on the right-hand side, $\mathrm{IDT_{PM}}$, can be used as the phase modulators. The bold arrows at the bottom indicate the vertical positions at which the samples were cleaved. For the experiments, the two long IDTs were employed. Supplementary Figure 3b shows a micrograph zoom-in to the acousto-optic interaction region of width l. $\mathrm{IDT_{PM}}$'s aperture is visible at the bottom of the image and seven IQPCs above. It is important to note that all individual IQPCs located on the sample can be modulated by this single IDT[9,10]. Supplementary Figure 3c-f show scanning electron microscope (SEM) images of key parts of a typical as-fabricated device: a zoom in to the output region of the second MMI (Supplementary Figure 3c), the cleaved end facet of an input port (Supplementary Figure 3d), an IDT and the two MZI arms (Supplementary Figure 3e) and waveguide section in a MZI arm (Supplementary Figure 3f).

Supplementary Note 2 – Experimental setup

A schematic of the experimental setup is shown in Supplementary Figure 4. The experiment is conducted in a closed-cycle helium cryostat at temperature of $T = 7$ K.

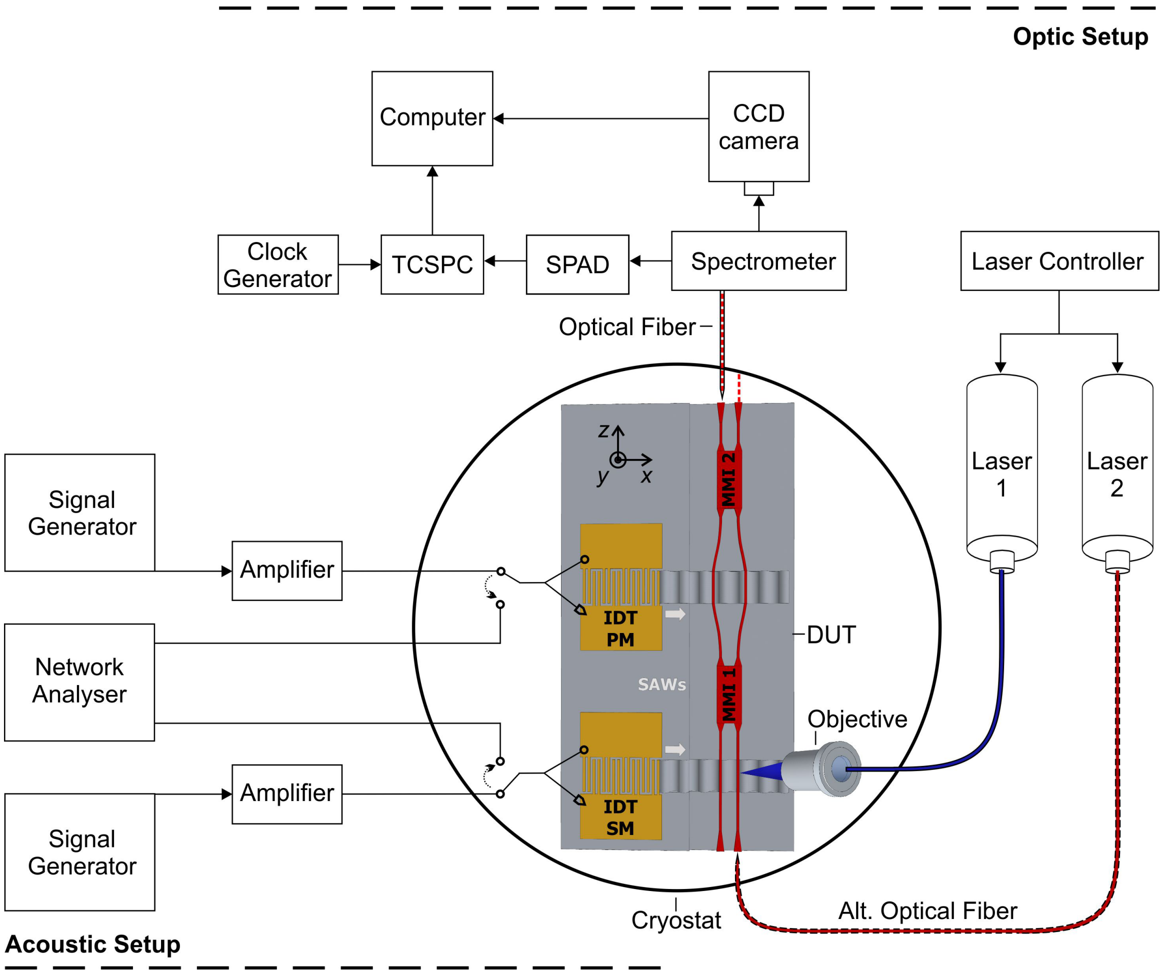

Supplementary Figure 4 – Experimental setup – Acoustic and optic setup of the measurement with a schematic of the device under test (DUT) located within the cryostat, sketched in the top view.

In the optical part, a laser (wavelength 660 nm) (Laser 1) is focused by an objective lens (NA = 0.81) to a diffraction limited spot on the device under test (DUT). It photo-generates carriers inside QDs which then emit single photons into the waveguide structure (colored red). At the top end of the DUT one lensed optical fiber is positioned to collect the light from the cleaved facet. For preliminary characterization a resonant laser (Laser 2) can be coupled in the IQPCs using a second lensed fiber on the opposite port. The collected signal was dispersed by a grating spectrometer and detected by a liquid nitrogen cooled charged-coupled device (CCD) camera or by single photon avalanche detectors (SPADs). The latter are connected to a time correlated single photon counting (TCSPC) electronics for time resolved detection.

The acoustic part comprises two RF signal generators connected to two IDTs (colored golden) to generate SAWs. The two generators are locked by a 10 MHz clock and referenced to the TCSPC to establish a common time base of experiments. For preliminary IDT characterization a network analyzer replaces the signal generators.

Supplementary Note 3 – Sample characterization

Supplementary Note 3.1 – Waveguide loss

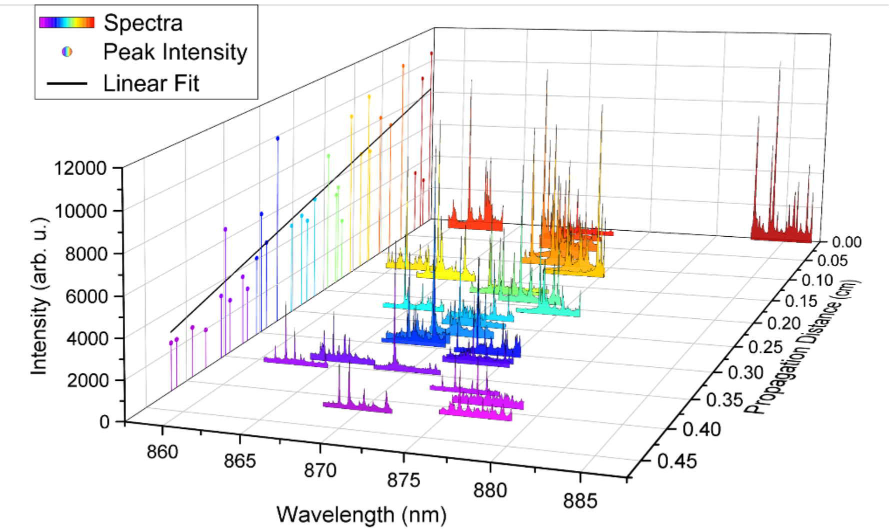

Supplementary Figure 5 – Propagation loss analysis – A series of QD spectra measured along the length of a single waveguide reveals the optical propagation loss.

Supplementary Figure 5 shows a series of PL spectra recorded from single QDs which are positioned along of a straight reference waveguide. From these data, we determine a propagation loss of (10.8±2.5) dB/cm, competitive with recently reported values for similar devices[11,12].

Supplementary Note 3.2 – SAW characterization

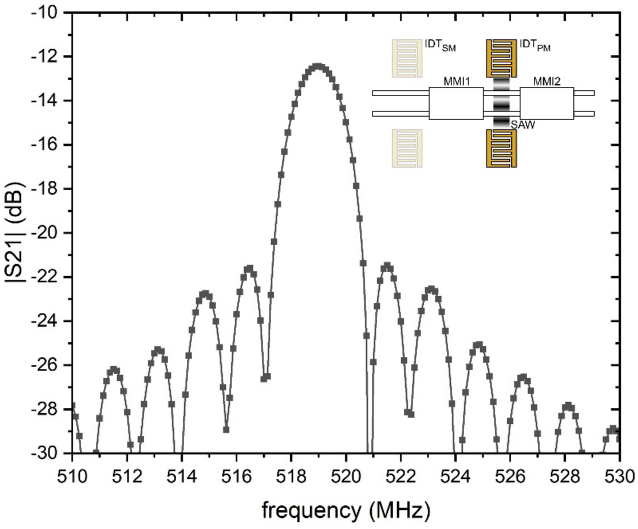

Supplementary Figure 6 – IDT transmission – Measured transmission (black symbols and line) for a split-2 finger IDT.

The fabricated IDTs were characterized employing vector network analysis. The measured transmission at room temperature with the sample being mounted on an impedance matched testboard is shown in Supplementary Figure 6 for the IDT device used as the PM. For the measurement, two IDTs on the same acoustic axis along [110] are used. At the resonance frequency of 519MHz the insertion loss of the delay line is 12.4 dB. As the delay line is symmetrical, this results in an electro-acoustic conversion efficiency of 24% for a single IDT. For low temperatures, the resonance frequency shifts to 525 MHz and the insertion loss increases to 28 dB. The increase of the insertion loss is attributed to an additional damping by the cryostat caused by longer rf-cables and a non-mode-matched electrical connection of the sample to the RF-cables due to spatial restrictions in the cryostat assembly. Overall, at low temperatures about 13% of the applied rf-power is transferred to the IDT electrodes. The electrical drive powers $P_{\mathrm{rf,PM}}$ and $P_{\mathrm{rf,SM}}$ specified in the main text are not corrected for this additional attenuation and correspond to the power applied to the cryostat connectors.

Supplementary Note 3.3 – Passive MZI coupling

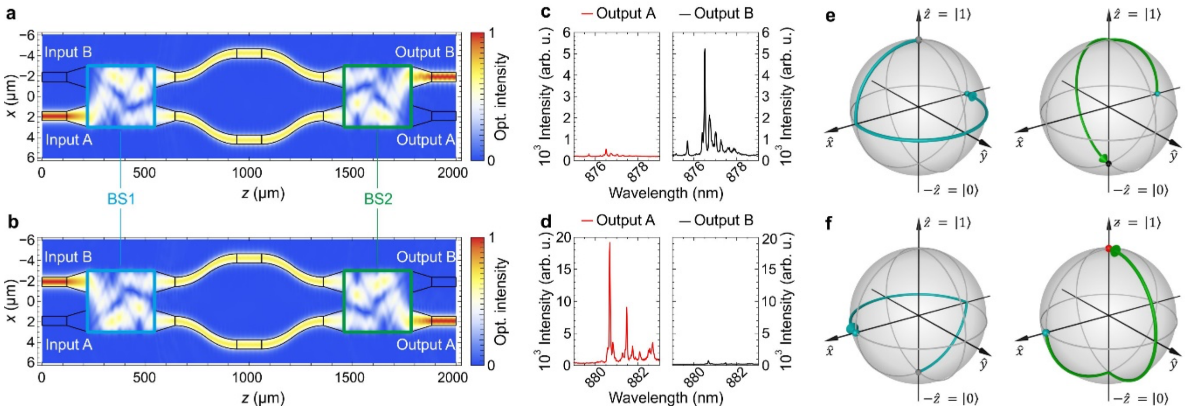

Supplementary Figure 7 – Passive device – BPM simulations of the light propagation through the passive device for light being generated in Input A (**a**) and Input B (**b**), respectively. Measurements of the static routing of the emission of two exemplary QDs from Input A to Output B (**c**) and Input B to Output A (**d**), respectively. The corresponding rotations of the qubit states on the Bloch sphere which are mapped from $|0\rangle \rightarrow |1\rangle$ (e) and $|1\rangle \rightarrow |0\rangle$ (f) are shown for each individual beam splitter (BS).

The unmodulated device is designed in the cross-coupling configuration shown in Supplementary Figures 7a and b, where the color-coded normalized optical field intensity is plotted as a function of position. The main panels show simulations of the light propagation obtained from beam propagation method (BPM) calculations. In these simulations, we assume an optical wavelength of $\lambda_{\mathrm{optical},A} = 876.5\ \mathrm{nm}$ (Supplementary Figure 7a) and $\lambda_{\mathrm{optical},B} = 880.5\ \mathrm{nm}$ (cf. Supplementary Figure 7b) which are matched to the two QDs' individual emission lines in Figs. S7c and d. In the two simulations, the optical signals are injected into Input A (cf. Supplementary Figure 7a) and Input B (Supplementary Figure 7b), respectively. As shown, the unmodulated, passive device is indeed designed in a cross-coupling configuration, i.e. Input A of the MZI and is coupled out via Output B. Conversely, Input B is coupled out via Output A. Next, we confirm the successful fabrication of such designed device using two single QDs located in different inputs and detecting the emission from the two outputs using a lensed optical fiber. Supplementary Figure 7c-d show measured emission spectra of two single QDs located in Input A and Input B, respectively. When the QD in Input A is excited (cf. Supplementary

Figure 7c), we detect a strong emission signal at Output B (right panel) while the intensity coupled out at Output A (left panel) is strongly suppressed. When switching to a QD in Input B (cf. Supplementary Figure 7d), the situation is reversed, and a strong signal is detected from Output A (left panel) and a weak signal from Output B (right panel). Without SAW-modulation, the fabricated MZI near-ideally maps an inversion of the input photonic qubits $|0\rangle \rightarrow |1\rangle$ and $|1\rangle \rightarrow 0\rangle$ with high fidelities of 0.90 and 0.96, respectively. Figs S6 e and S6 f show the corresponding qubti rotations on the Bloch sphere.

Supplementary Note 3.4 – Simulation of the MZI switching dynamics

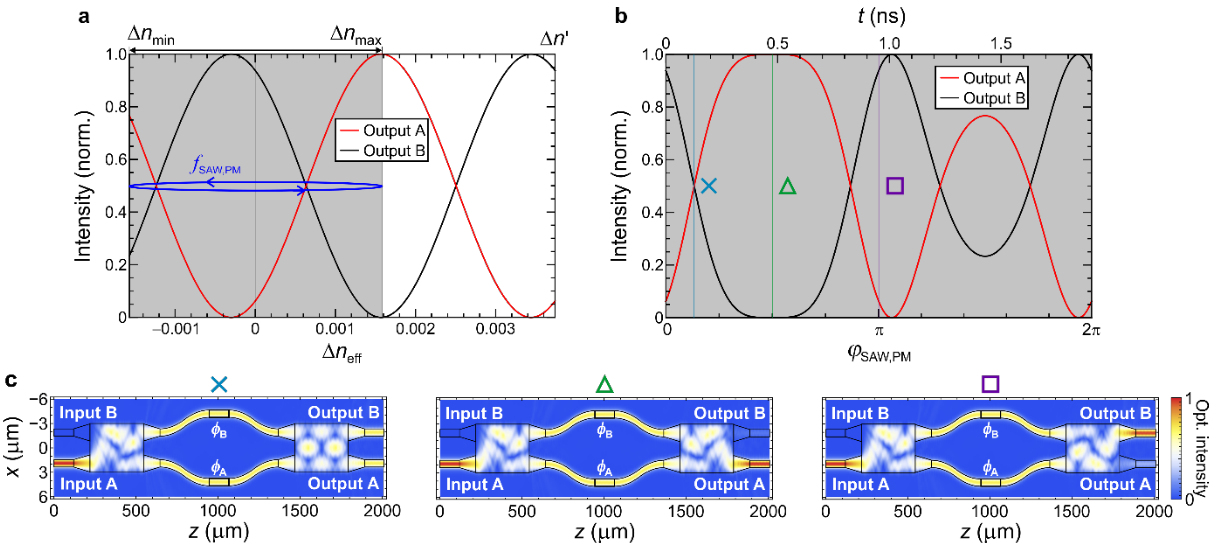

Supplementary Figure 8 – Time resolved optical transmission pattern analysis – (**a**) Calculated optical transmission of the acoustically modulated MZI for a linearly increasing refractive index amplitude in the acousto-optic interaction region. (**b**) Optical transmission of the acoustically modulated MZI as a function of acoustic phase and time. (**c**) Optical field distribution in the device at three distinct phases of the acoustic cycle.

Supplementary Figure 8a shows the calculated optical transmission intensity when the refractive index difference Δn_{eff} between the two interferometer arms in the acousto-optic interaction region increases linearly. The grey background area marks the modulation achieved in the experiment (cf. Figure 2c of the main manuscript) with $\Delta n_{\mathrm{max}} = -\Delta n_{\mathrm{min}}$ marking the range of the effective refractive index change Δn_{eff} that causes the optical phase shift $\phi_{A/\mathrm{B}}$. Here, Δn_{s} is the static refractive index difference between the MZI arms which causes the device characteristic optical transmission pattern observed in the experiment. $\Delta n'$ is the increment of refractive index change for which the default, cross coupling configuration of the device is restored. Using these parameters, the time dependent transmission can be expressed as

$$I_{\mathrm{opt}\pm}(t) = \frac{1}{2}[1 \pm \cos\{2\pi(\Delta n_{\mathrm{eff}} \sin(2\pi f_{\mathrm{SAW,PM}} t) + \Delta n_{\mathrm{s}}/2)/\Delta n'\}]. \quad (1)$$

Here, $I_{\mathrm{opt}\pm}(t)$ is the intensity of the time dependent optical transmission of the acoustically tuned device[9] and is plotted in Supplementary Figure 8b for both Outputs B with $I_{\mathrm{opt}-}(t)$ and Output A with $I_{\mathrm{opt}+}(t)$ using $\Delta n_{\mathrm{eff}} = \Delta n_{\mathrm{max}} = 0.00157$.

Supplementary Note 3.5 – Acousto-optic spectral tuning of QDs

We analyze the spectral modulation of the emission of a single QD in the input arms using IDT_{SM}. As shown in the schematic in Supplementary Figure 9a, a resonant radio frequency signal ($P_{\mathrm{rf,SM}} = 22\ \mathrm{dBm}$ and $f_{\mathrm{SAW,SM}} = 524.12\ \mathrm{MHz}$) is applied to IDT_{SM} while no signal is applied to IDT_{PM}. A single QD is excited in Input A and its emission is detected via Output B. Supplementary Figure 9b shows the color-coded normalized emission intensity of the QD as a function of wavelength (vertical axis), phase and time (horizontal axis) over two acoustic cycles. We detect a clear sinusoidal spectral modulation with the expected period of $T_{\mathrm{SAW,SM}} = 1.905\ \mathrm{ns}$. The dependence of the measured amplitude of this modulation $\Delta\lambda$ (symbols) is plotted in double-logarithmic representation as a function of $\sqrt{P_{\mathrm{rf,SM}}}$ in Supplementary Figure 9c. The latter is proportional to the amplitude of the SAW, $A_{\mathrm{SAW}} \propto \sqrt{P_{\mathrm{rf,SM}}}$. The data are in good agreement with a power law dependence $\Delta\lambda \propto \left(\sqrt{P_{\mathrm{rf,SM}}}\right)^m$. From the best fit (red line) we obtain $m = 1.3 \pm 0.1$, close to $m = 1$ expected for deformation potential coupling[13,14]. The measured broadening is given in terms of energy $2\Delta E_{\mathrm{DP}}$ on the right-hand-side scale. These data validate the dynamic spectral tuning of an integrated single photon emitter as the first key component of our IQPC device.

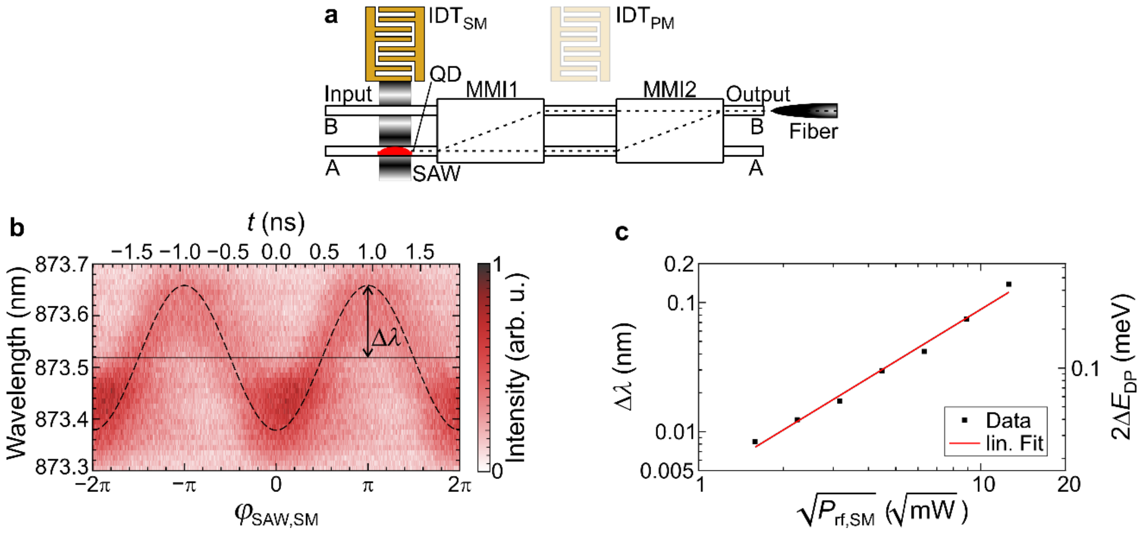

Supplementary Figure 9 – Spectral modulation of waveguide coupled quantum dot emission – (a) Schematic representation of the experimental configuration: the optical transition of a single QD in Input A is modulated by a SAW generated by IDT_{SM} (kept on, illustrated in bright colors), the response of the MZI is static (IDT_{PM} is turned off, illustrated in shade) as the photons passes through. The spectrally modulated emission is collected via Output B. (**b**) Measured time-dependent emission spectra of a single QD ($P_{\mathrm{rf,SM}} = 22\ \mathrm{dBm}$ and $f_{\mathrm{SAW,SM}} = 524.12\ \mathrm{MHz}$) show a clear modulation of the emitted wavelength. (**c**) Spectral broadening of the QD response as a function of the applied power $\sqrt{P_{\mathrm{rf,SM}}}$, which is proportional to the SAW amplitude.

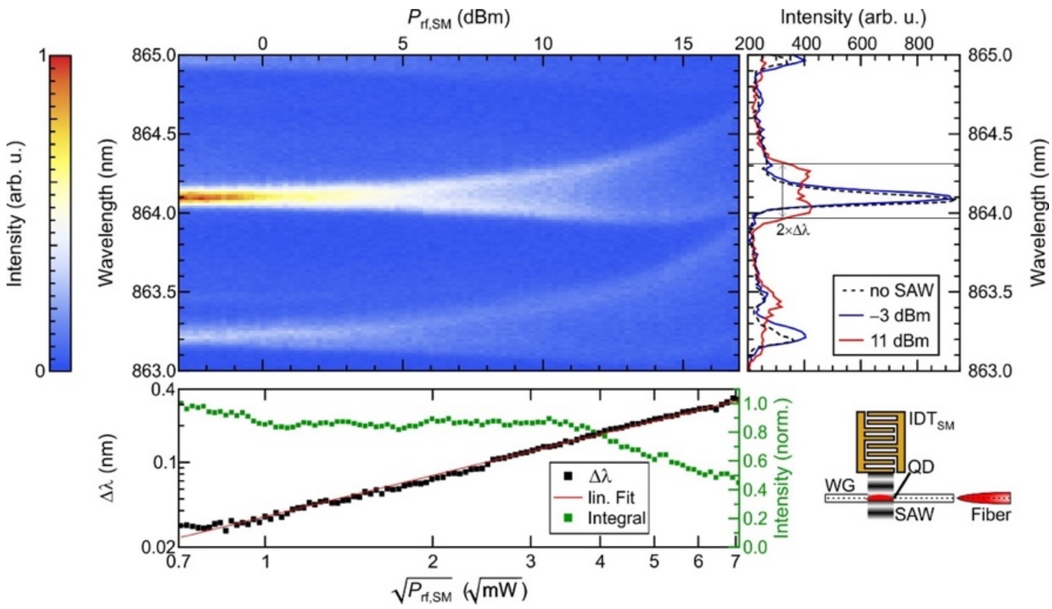

Supplementary Figure 10 – Spectral broadening in a straight waveguide – Spectral broadening of photons coupling to a single straight waveguide as a function of the applied power $P_{\mathrm{rf,SM}}$ in the main panel and $\sqrt{P_{\mathrm{rf,SM}}}$ in the lower panel. The right-hand panel compares two selected, modulated spectra to the unmodulated case.

Supplementary Figure 10 shows measurement data from an acoustically modulated QD positioned in a single straight waveguide structure. For comparison, the false color plot shows spectral broadening (vertical axis) of the QD response for increasing acoustic powers (horizontal axis). Again, a temperature dependent shift of the emission spectra for high modulation powers is also observed as discussed above and is discussed in Supplementary Note 3.6.
The spectrum for low acoustic modulation (-3 dBm, blue line), moderate modulation (11 dBm, red line) and the unmodulated case (dashed line, intensity not to scale) are highlighted in the panel to the right. In the lower panel the spectral broadening is again plotted in double-logarithmic representation as a function of $\sqrt{P_{\mathrm{rf,SM}}}$ in units of $\sqrt{\mathrm{mW}}$. Through a best fit to the data, we obtain $m = 1.13 \pm 0.01$. The green symbols show the integrated optical intensity of the main signal decreases to approx. 55% when increasing the RF power to 17 dBm.

Supplementary Note 3.6 – Temperature dependent effects

Quantum Dots

In Supplementary Figure 11 we plot the energy shift of three self-assembled (In,Ga)As QDs as a function of the temperature of the cryostat cold finger (symbols). The measurement is conducted on a reference sample without IQPCs. The emitted photons are collected through the same objective through which the laser signal is focused on the sample to excite the QDs' emission. The corresponding lines are extrapolations to the experimental data[15]. Using these extrapolations, we obtain a temperature increase to $\approx 55\ \mathrm{K}$ for the measured spectral shift in Figure 2b of the main paper. This spurious heating can be suppressed by pulsed SAW excitation schemes[15].

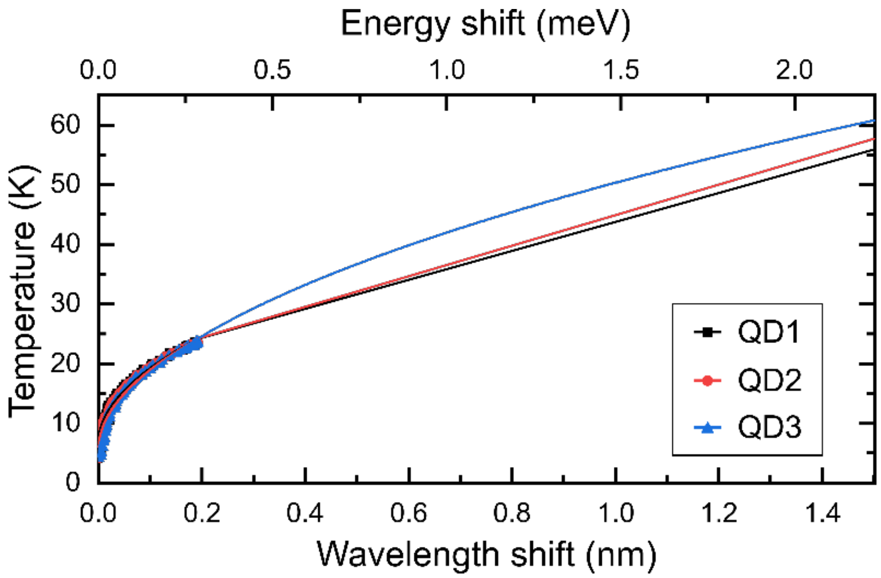

Supplementary Figure 11 – Temperature dependent spectral shift – Temperature dependence of the spectral response for three QDs (symbols) and fit to the data (lines).

Waveguide transmission

We assessed the dependence of the waveguide transmission on the applied RF power at room temperature and low temperature (7 K).

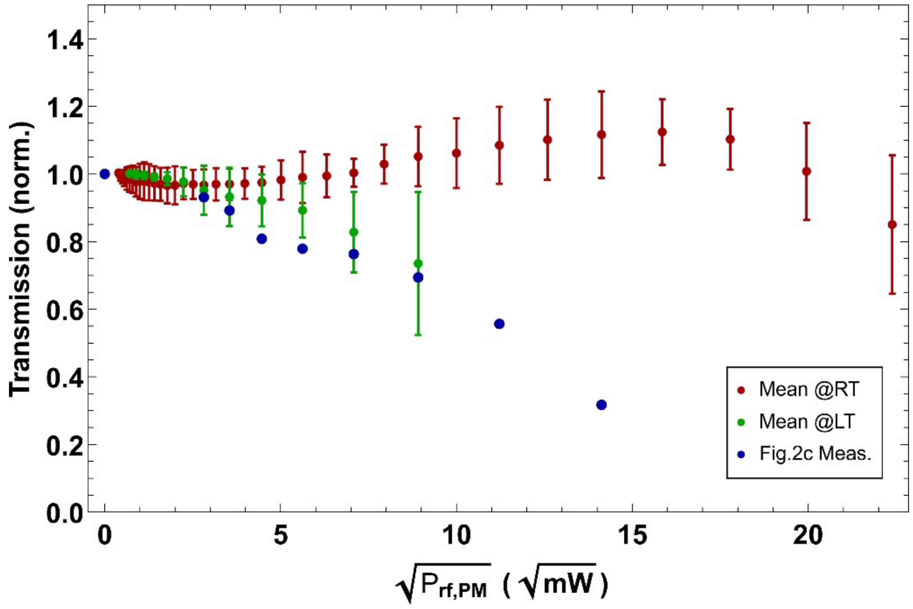

Supplementary Figure 12 – Impact of SAWs on the photonic transmission – Mean total photonic transmission measured for experiments at room temperature (RT) and at low temperatures (LT) with a SAW applied to modulate the phase.

Supplementary Figure 12 shows the mean of the photonic transmission as a function of applied RF power measured for three devices with MMI lengths of 300.5 µm, 314 µm and 326 µm each and at different optical wavelengths between 910 nm and 970 nm (Laser-2 in Supplementary Figure 4) at room temperature (red, 6 devices). At low temperature (green) the wavelengths of photons emitted by QDs varies from 873 nm to 913 nm between seven individual measurement series. The error bars for the mean transmission values for each SAW power measured are calculated as 1σ. These data

show no significant optical transmission loss induced by the SAW generated by IDT_{PM} at RT. At low temperatures, 7 K) a pronounced reduction is observed. This reduction may arise from a reduced emission intensity due to the elevated temperature. The blue symbols show complementary data from the measurement series analyzed in Figure 2c of the main manuscript which shows the same trend. Moreover, the data presented in Supplementary Figure 10 shows a similar reduction of the emission intensity at strong spectral drive supporting our assumption that the reduction of the signal intensity arises from heating.

Supplementary Note 4 – Second order autocorrelation function experiments

Supplementary Note 4.1 – Second order correlation function of unmodulated QD

Here, we present the measurement of the second order correlation function of a QD emitting photons into a straight waveguide. The QD is located approximately 425 µm from the sample edge where the lensed fiber is located for signal reading, as sketched in the inset of Supplementary Figure 13a. The main panel of Supplementary Figure 13a. shows the recorded time-integrated PL spectrum with the selected emission line marked by the blue arrow. The measured the second order correlation function is shown in Supplementary Figure 13b. The anti-bunching dip at $\tau = 0$ is clearly pronounced, proving the single photon nature of the signal. From a best fit to the data (see Eq. 2 below) we get $g_A^{(2)}(0) = 0.48 \pm 0.03$. Since no SAW is applied, the FFT analysis displayed in the inset of Supplementary Figure 13b does not show a peak at any particular frequency in contrast to the data in the main paper, in which there is a clear peak at the applied SAW frequency.

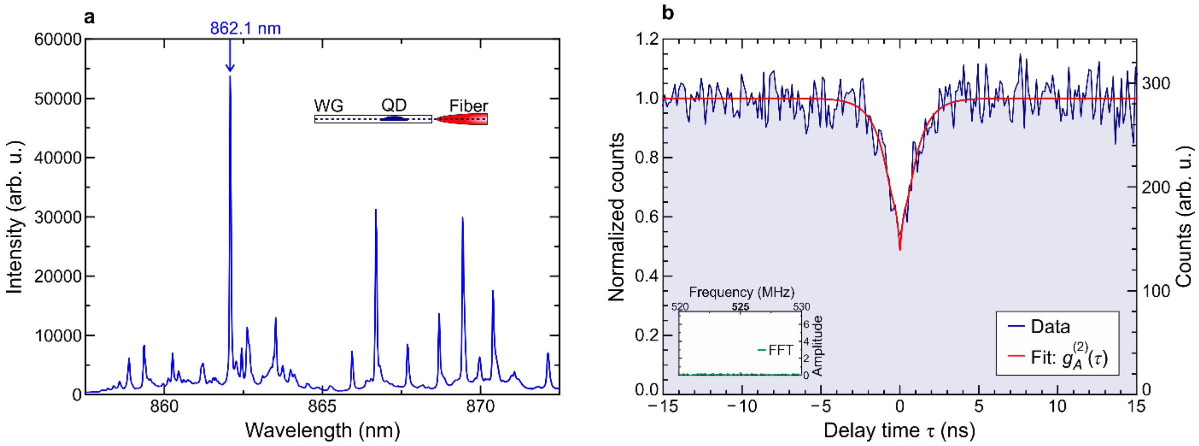

Supplementary Figure 13 – Anti-bunching in absence of acoustic excitation – (**a**) Spectrum of a QD located in a straight waveguide. (**b**) Measured second order correlation function for the unmodulated QD emission.

Supplementary Note 4.2 – Intensity modulation of the two QDs selected for $g^{(2)}$ experiments

Supplementary Figure 14 shows pre-characterization data of the QDs used for the measurements of the second order autocorrelation functions $g^{(2)}(\tau)$ in Figure 3 of the main paper. Supplementary Figure 14a presents the experimental configuration with one QD in Input WG-A (red) and another one in Input WG-B (black). The measured signals are collected through Output WG-B. Supplementary Figure 14b shows the measured PL- spectra of both QDs with the emission lines used for measuring $g^{(2)}(\tau)$ marked. Supplementary Figure 14c shows the time resolved measurement of the intensities of the two QDs with a SAW generated by IDT_{PM} under same conditions as $g^{(2)}(\tau)$ was measured: the trace for Input A (red line) corresponds to Fig. 3a and the measurement for Input B (black line)

corresponds to Figure 3b in the main paper, respectively. As expected, the emission of the QD in Input-B (black) is normally off and periodically routed to Output-B

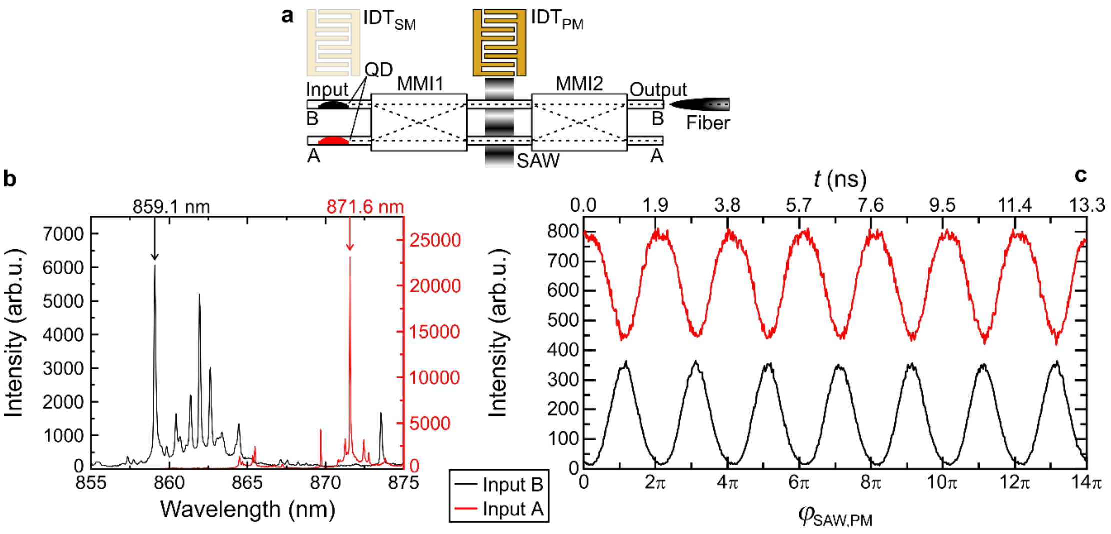

Supplementary Figure 14 – Characterization of the QDs of the $g^{(2)}(\tau)$ measurements – (a) Sketch of the measurement configurations. (**b**) Spectra emitted by the QDs corresponding to the $g^{(2)}(\tau)$ measurements for the device presented in Fig. 3a (Input A) and Fig. 3b (Input B) of the main manuscript. (**c**) Measured phase and time resolved optical transmission from both QDs.

Supplementary Note 4.3 – Fit functions

The function used on the data collected from the second order correlation measurements of the device in modulated, cross-coupling configuration presented in Figure 3a of the main manuscript and for the unmodulated device shown in Supplementary Figure 13b is given by

$$g_A^{(2)}(\tau) = 1 - (a_0 e^{\left(-\frac{|\tau-\tau_0|}{t_d}\right)} + a_1 e^{\left(-\frac{|\tau-\tau_0|^2}{2t_1{}^2}\right)}). \quad (2)$$

Here, t_1 is a constant accounting for the SPAD time resolution of 300 ps, t_d is the anti-bunching time constant, a_0 and a_1 are amplitudes and τ_0 accounts for a finite shift in delay time.

The function used to fit the data of the modulated device in bar-coupling state shown in Fig. 3b of the main manuscript was

$$g_B^{(2)}(\tau) = a_0 \sin(2\pi(\tau-\tau_0)/T_0) - a_1 e^{\left(-\frac{|\tau-\tau_0|}{t_1}\right)}. \quad (3)$$

Here, T_0 is the SAW modulation period.

Supplementary Note 5 – Wavelength selective routing: complementary measurements and analysis

Direct coupling and cross-coupling configuration

Figures 4 c-f of the main paper show wavelength selective dynamic routing for the direct coupling configuration from Input B to Output B. Here, we compare the full data for both coupling configurations shown in Supplementary Figure 15 a. The data for direct coupling via Output B (identical to those in the main paper) are plotted in Supplementary Figures 15 b-e. The data for cross coupling

in Supplementary Figure 15 f-i is perfectly anti-correlated. This anti-correlation is corroborated further by extracting the intensities at the minimum ($\lambda_{\min}$) and maximum ($\lambda_{\max}$) wavelengths of the spectral modulation from Output A (red) and Output B (black) in Supplementary Figure 15 j and k, respectively.

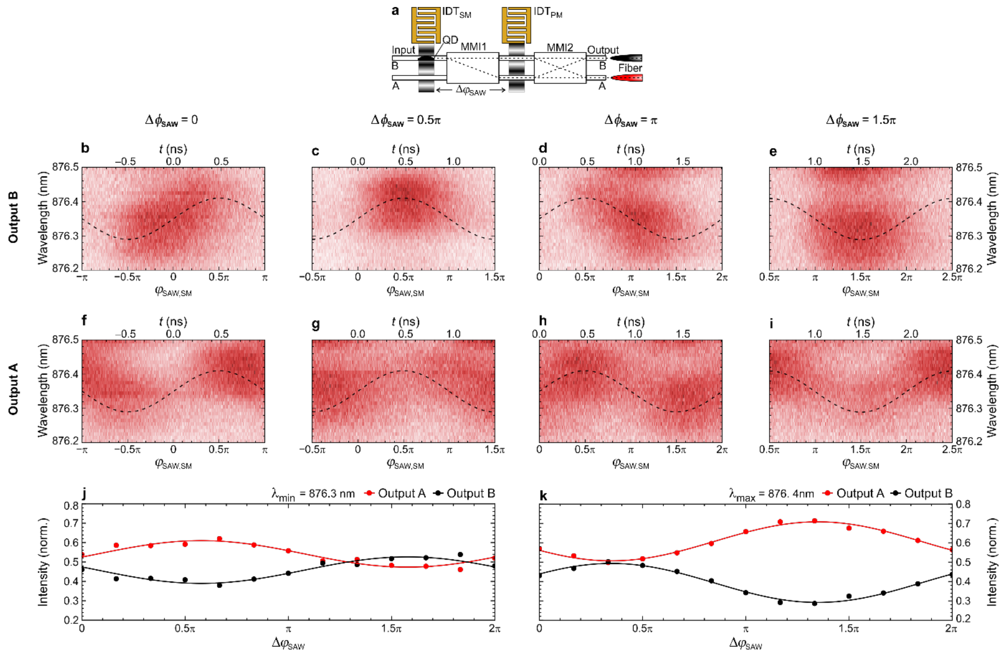

Supplementary Figure 15 – Wavelength selective dynamic routing in bar and cross-coupling configuration – (**a**) Schematic of the experimental configurations. Phase and time dependent emission spectra of the QD in Input B measured from Output B (**b-e**) and Output A (**f-i**) at four distinct phases $\Delta\phi_{\mathrm{SAW}}$. Routing of the emission wavelength $\lambda_{\min}$ (**j**) and $\lambda_{\max}$ (**k**) in both Outputs A and B.

Suppelmentary References

1. Tamir, T. *Integrated Optics*. vol. 7 (Springer Berlin Heidelberg, 1975).

2. Gehrsitz, S. *et al.* The refractive index of $Al_xGa_{1-x}As$ below the band gap: Accurate determination and empirical modeling. *Journal of Applied Physics* **87**, 7825–7837 (2000).

3. Palik, E. D. Gallium Arsenide (GaAs). in *Handbook of Optical Constants of Solids* 429–443 (Elsevier, 1997). doi:10.1016/B978-012544415-6.50018-2.

4. Talghader, J. & Smith, J. S. Thermal dependence of the refractive index of GaAs and AlAs measured using semiconductor multilayer optical cavities. *Applied Physics Letters* **66**, 335–337 (1995).

5. Crespo-Poveda, A. *et al.* Semiconductor optical waveguide devices modulated by surface acoustic waves. *Journal of Physics D: Applied Physics* **52**, 253001 (2019).

6. Soldano, L. B. & Pennings, E. C. M. Optical multi-mode interference devices based on self-imaging: principles and applications. *Journal of Lightwave Technology* **13**, 615–627 (1995).

7. Okamoto, K. *Fundamentals of Optical Waveguides*. (Elsevier, 2006). doi:10.1016/B978-0-12-525096-2.X5000-4.

8. de Lima, M. M., Beck, M., Hey, R. & Santos, P. V. Compact Mach-Zehnder acousto-optic modulator. *Applied Physics Letters* **89**, 121104 (2006).

9. Beck, M., de Lima, M. M. & Santos, P. V. Acousto-optical multiple interference devices. *Journal of Applied Physics* **103**, 014505 (2008).

10. Beck, M. *et al.* Acousto-optical multiple interference switches. *Applied Physics Letters* **91**, 061118 (2007).

11. Schnauber, P. *et al.* Deterministic Integration of Quantum Dots into on-Chip Multimode Interference Beamsplitters Using in Situ Electron Beam Lithography. *Nano Letters* **18**, 2336–2342 (2018).

12. Reithmaier, G. *et al.* On-Chip Generation, Routing, and Detection of Resonance Fluorescence. *Nano Letters* **15**, 5208–5213 (2015).

13. Weiß, M. & Krenner, H. J. Interfacing quantum emitters with propagating surface acoustic waves. *Journal of Physics D: Applied Physics* **51**, 373001 (2018).

14. Weiß, M. *et al.* Dynamic Acoustic Control of Individual Optically Active Quantum Dot-like Emission Centers in Heterostructure Nanowires. *Nano Letters* **14**, 2256–64 (2014).

15. Weiß, M. *et al.* Surface acoustic wave regulated single photon emission from a coupled quantum dot–nanocavity system. *Applied Physics Letters* **109**, 033105 (2016).
